# Prospects of labour migration pressure in Algeria, Morocco, Tunisia and Turkey

**DOI:** 10.1186/s41118-016-0015-x

**Published:** 2016-12-02

**Authors:** W. G. F. (George) Groenewold, J. A. A. (Joop) de Beer, H. A. G. (Helga) de Valk

**Affiliations:** Netherlands Interdisciplinary Demographic Institute (NIDI/KNAW/RUG), Lange Houtstraat 19, 2511 CV The Hague, The Netherlands

## Abstract

Gaining control over refugee flows and undocumented migrants currently dominate the media and political arenas in Europe. Underlying driving and enduring forces, such as employment-related migration pressure, tend to be relegated to the background. In this article, we explore migration pressure prospects up to 2035 in four countries with a tradition of emigration to Europe: Algeria, Morocco, Tunisia and Turkey. More specifically, we first derive a simple decomposition model based on the relationship between working-age population (WAP) growth and growth of gross domestic production (GDP) and worker productivity (GDP/*W*). From this model, we derive an indicator of migration pressure: size of the non-employed population in a country. This model is then used as framework for deriving storylines for three different scenarios of economic and demographic change up to 2035. Subsequently, storylines are operationalized, leading to scenario estimates of migration pressure up to 2035. The implications of the results are then discussed. Time series of macro-level economic and demographic data are used to underpin scenario assumptions.

Scenario results suggest that in all countries employment ratios are expected to increase, but only in Tunisia is the size of the non-employed population—our indicator of migration pressure—expected to decline, irrespective of the scenario. Depending on the scenario, migration pressure remains high in Turkey and Morocco and may even become somewhat higher. The general conclusion is that in the long term, after 2035, labour migration pressure can be expected to decrease because the growth and size of the working-age population is decreasing while employment ratios are rising.

## Introduction

Today’s debates in the media and political arenas in countries of the European Union (EU) are dominated by how to gain control over rising numbers of refugees and uninvited labour migrants from countries in Africa and Asia. Recent deliberations about visa-free travel for Turkish citizens entered the debate because it may lead to new flows of employment-seeking Turkish citizens to the EU. This focus on the management of and control over immigration and migrant-integration tends to overshadow the driving and enduring forces underlying migration in the countries of origin, such as un- or underemployment. This is the focus of our article, whereby we explore current conditions and future prospects of employment-related migration pressure in four countries in the Mediterranean region with a tradition of emigration to Europe: Algeria, Morocco, Tunisia and Turkey. Although immigrants to the European Union come from many countries, the great majority of non-western immigrants come from these four countries only (e.g. Fargues 2005).

During the 1950s and 1960s, these countries became the main countries of origin of immigrants to Europe. Poverty and high rates of unemployment were push factors while high demand for low-skilled labour was the main pull factor. Most labour migrants, almost exclusively men, decided to stay in destination countries and reunify with spouses, family and relatives. By 2005, over six million people had moved from countries in the South and Southeast Mediterranean region to the EU. About 90% of these immigrants came from the three Maghrebi countries and Turkey. While Maghrebi migrants mainly moved to France, Spain, Italy and The Netherlands, Turkish migrants mainly moved to Germany, The Netherlands, Austria and France (Fargues 2005; OECD 2015).

Since the 1980s, demand for non-EU low-skilled labour immigrants waned, while demand for jobs has remained high in these origin countries, despite considerable economic growth. For instance, between 2000 and 2015, gross domestic product (GDP) growth was in the range of 3 to 6% annually but this was mainly due to increase in the value of output produced by existing workers—our proxy for worker productivity—than to the increase of numbers of workers (ILO 2015; IMF 2015). Furthermore, these countries realized growth rates of working-age populations in the age range 15 to 65 years which outperformed economic growth rates (UNPD 2016). This contributed to increase of welfare disparities with EU neighbours and pressure to emigrate (e.g. Castles et al. 2014; EC 2011; Schiffbauer et al. 2015). To illustrate, in 2015, per capita GDP in Algeria was about $13,823 (PPP, constant 2011 international dollars ($)), $7,361 in Morocco, $10,726 in Tunisia and $18,959 in Turkey. In the main destination countries, it was $44,053 (Germany), $46,374 (The Netherlands) and $37,306 (France) (World Bank 2016). Labour emigration to the EU has increasingly become constrained by stricter EU immigration policies, following concerns about national security, immigrant integration and social cohesion (e.g. Carrera et al. 2012)

This raises the question about what the future might bring in terms of migration pressure in these four countries. EU governments expect that pressure will further increase by pointing at increasing numbers of citizens and transit migrants attempting to enter the EU illegally in search of work or refuge (e.g. Collett 2013; Fergusson 2014). Qualitative scenarios of the Organisation for Economic Co-operation and Development (OECD) up to 2035 suggest that labour migration pressure will rise due to overcrowding of local labour markets, despite modest growth of job opportunities (OECD 2009). Others foresee that in the long-term labour migration pressure in these four countries will dissipate because there are signs that population growth rates are declining in these countries and that these countries may increasingly become countries of immigration of residents of other countries in Africa and Asia (e.g. De Haas 2011). So far, these views have not been underpinned by quantitative exploratory research.

In this article, our objectives are (1) to identify a macro-economic and demographic decomposition model as framework for deriving economic-demographic scenarios up to 2035 for four study countries and (2) to explore what the implications of scenario results are for employment-related migration pressure.

## Conceptualization and measurement

A plethora of macro- and micro-level factors give rise to international migration, such as psychological, social, economic, political and environmental factors in countries of origin and destination (e.g. Castles et al. 2014). Depending on the setting, income and employment differences between countries and regions can be important in explaining international migration, such as in the case of our study countries. In the academic literature, several theories confirm that spatial differences in employment are important to international migration. For instance, dual and segmented labour market theories posit that migration is mainly the result of labour needs in destination countries arising from upward job mobility of the resident population, away from insecure, low-quality and low-income jobs. Immigrants are expected to fill such positions. People migrate because they are ‘pulled’ by demands and opportunities in foreign labour markets. Neoclassical economic theory adds a micro-level decision-making perspective by arguing that persons migrate because financial returns to their educational attainment and occupational skills are higher elsewhere. The new economics of migration theory expounds this view by pointing to the importance of the household as the decision-making unit. In this context, decisions are taken as to who migrates and what is expected of the migrant. Migration is just one of several income risk-aversion and risk-diversification strategies households apply to survive. Migrants are expected to remit part of their earnings leading some receivers to migrate too. World systems theory places micro-level migration decision-making in the wider context of societal-level factors. For instance, interacting labour markets may lead to the transfer of conventions about employer-employee relations from one country to another, pushing certain people out of work in one country and increasing demand for their labour in another country, leading to migration (Castles et al. 2014; Van Dalen et al. 2005a).

At the macro-level, pressure to migrate is often defined rather loosely and differently, such as in terms of high demographic growth, low welfare and wellbeing or lack of employment (Castles et al. 2014). Some authors are more specific in their definition. For instance, Schaeffer (1993) defines migration pressure in a country in terms of the volume of long-term demand for income-earning opportunities abroad. This pressure is influenced by people’s expected economic, legal and social status abroad, costs of migration and other characteristics in destination countries. Potential migrants compare destination countries and country of residence regarding such features. Straubhaar (1993) defines migration pressure as excess supply of migration-willing people in origin countries relative to total demand for immigrants in destination countries. In this definition, great importance is attributed to the effect of migration policies in destination countries on migration pressure in origin countries.

Migration pressure as defined by Bruni and Venturini (1995), the one adopted in this article, is particularly useful to our study. Contrary to the previous two definitions, focus is almost entirely on conditions in countries of origin. Migration pressure is defined as excess domestic labour supply in the presence of *negative* per capita income differences with other countries. The latter means that labour migrants only move to countries where employment and income prospects are higher.

Bruni and Venturini (1995, pp. 380–381) make a distinction between migration pressure and actual migration. The former is defined as a macro-level phenomenon and the latter as a micro-level phenomenon. More specifically, migration pressure is indicated by the presence of *excess* labour supply, which is measured in terms of the size of the non-employed population of working age. Migration pressure increases when numbers of non-employed persons decrease and vice versa. Migration pressure is to be interpreted as *potential* migration at the macro-level. However, actual migration is defined at the micro-level. It is the *propensity* of an individual to migrate, i.e. the probability that an individual willing to migrate will indeed migrate. This probability depends on individual-level socioeconomic and demographic person characteristics and conditions of the personal context, including perceptions about employment and income abroad and barriers to migration such as restrictive immigration policies. Actual migration is ‘release’ of migration pressure, whereby the volume and direction of migration are conditioned by the extent of negative income differentials with other countries. By making a distinction between migration pressure and actual migration, Bruni and Venturini (1995) avoid a potential circularity problem in the analysis of population projections in that assumptions about future actual (net) migration at the micro-level influence results and conclusions about migration pressure at the macro-level.

This conceptualization of migration pressure and actual migration is applicable to situations in poor and wealthy nations. In countries where income is low and unemployment benefits and social security systems absent, increase of excess labour supply can be considered as a thermometer or early warning system for rising emigration *intentions*. Unemployment and low income undermine the quality of life in households, especially in the long term. This puts pressure on household members to migrate to places where employment and income prospects are brighter. In wealthier nations, increase of excess labour supply generally does not lead to migration but only to a rise of the non-employed population—and unemployment rates—because income prospects elsewhere are mostly lower and because loss of employment is to some extent financially compensated by the unemployment and social security benefit system (Bruni and Venturini 1995, p. 380).

Bruni and Venturini (1995, p. 382) also argue that the employed and underemployed frequently are the ones migrating because such persons are responsible for sustaining the lives of household members staying behind. Migration pressure increases if numbers of non-employed and financially dependent persons in the personal network increase. This is confirmed by empirical studies (e.g. Esipova et al. 2011). Therefore, at the macro-level, increase of the non-employed population in a country can be taken as proxy for the inability of a country’s domestic economy to provide sufficient income-earning employment to its working-age population and for a rise of migration pressure at the macro-level.

Change in migration pressure—size of the non-employed population—is determined by (1) change in the employment ratio and (2) change in working-age population size. The numerator of the employment ratio—numbers of employed persons—is determined by economic factors, i.e. gross domestic product and output per worker (e.g. Gutierrez et al. 2007; Shorrocks 1999; World Bank 2014a, 2014b). Output per worker features as proxy for worker productivity and includes aspects of innovation and technology, capital and labour. The denominator of the employment ratio—the working-age population—is determined by demographic factors (e.g. fertility, mortality, migration). The following parsimonious decomposition model summarizes and helps quantifying the interrelatedness of all of these factors:

1 $$ {\mathrm{GDP}}_t = \frac{{\mathrm{GDP}}_t\ }{W_t}\times \frac{W_t}{{\mathrm{WAP}}_t}\times {\mathrm{WAP}}_t $$

Essentially, the model decomposes gross domestic product (GDP) into contributions from output per worker (GDP/*W*), the employment ratio (*W*/WAP) and the size of the working-age population (WAP). We use this model as analytical framework for deriving economic and demographic scenarios for the period 2015–2035 and for assessing migration pressure change implied by the scenario results. To do so, the above decomposition model must be restated in terms of (1) *change* of the employment ratio and (2) *change* of the size of the non-employed population.

First, restatement in terms of change of the employment ratio is accomplished by taking natural logarithms and rearrangement of terms in Eq. (1), leading to the following model for measuring change of the employment ratio:

2 $$ \Delta \frac{W_t}{{\mathrm{WAP}}_t}=\left({\Delta \mathrm{GDP}}_t - \Delta \frac{{\mathrm{GDP}}_t}{W_t}\right)-{\Delta \mathrm{WAP}}_t $$

where ∆*X* equals the difference in the logarithm of *X* between 2 years. More specifically, Eq. (2) conveys that the rate of change of the employment ratio depends on rates of change in GDP, worker productivity and the working-age population. The implication is that, if the net (combined) rate of change of the two economic factors exceeds the rate of change of the demographic factor, then the employment ratio increases so that a larger share of the working-age population is employed. Thus, if

3 $$ \left({\Delta \mathrm{GDP}}_t - \Delta \frac{{\mathrm{GDP}}_t}{W_t}\right)>{\Delta \mathrm{WAP}}_t $$

Second, restatement in terms of change of the non-employed population is influenced by change in the aforementioned employment ratio *as well as* by change in the size of the working-age population, that is,

4 $$ {\Delta \mathrm{N}\mathrm{E}}_t = {\Delta \mathrm{WAP}}_t+\Delta \left(1-\frac{W_t}{{\mathrm{WAP}}_t}\right) $$

More specifically, the size of the non-employed population only starts declining after decrease of the *non-*employment ratio becomes larger than the increase of the working-age population; thus, if

5 $$ \Delta \left(1-\frac{W_t}{{\mathrm{WAP}}_t}\right)<-{\Delta \mathrm{WAP}}_t $$

Thus, to explore migration pressure change during the 2015–2035 period involves exploring future change in employment ratios and change in size of the non-employed population, whereby we have to factor in the force of demographic growth embedded in the population.

In terms of our analytical framework—decomposition model (1)—migration pressure change can be explored after developing scenario assumptions about future change in the model variables gross domestic production (GDP) and output per worker (GDP/*W*), and about the determinants of working-age population (WAP) change (i.e. fertility, mortality and actual migration). Below, we describe the storylines of three distinct economic-demographic pathways to the future, including operationalization and methodological aspects.

## Three economic-demographic scenarios

### Scenario storylines

The business-as-usual scenario assumes that recent trends in demographic and economic indicators for the four countries continue in future. The open economies scenario assumes that countries develop as open societies participating in a global economy without barriers to production, trade and international migration. The closed economies scenario, a kind of anti-globalization scenario, assumes that countries move away from international cooperation in favour of protectionism and restrictions regarding the movement of capital, goods and people.

More specifically, the *business-as-usual scenario* assumes a continuation of past trends (2000–2015) in terms of GDP and output per worker and production and trade characterized by ad hoc styles of cooperation between countries in the region and with the EU (e.g. EC 2011). The scenario assumes no further breakthrough in political, social, technological and cultural changes in these countries, while innovation and adoption of new technologies in production, management, infrastructure and administration will be at levels observed during the 2000–2015 period. By international standards, GDP and output per worker remain at relatively low levels, notably in Morocco, Algeria and Tunisia (ILO 2015). During the period 2000–2015, average annual GDP growth was in the range of 3.2% (Tunisia) and 4.5% (Morocco), while growth of output per worker was in the range of 2 and 3%, respectively, except in Algeria where for many years output per worker stagnated. The value of our proxy for worker productivity—GDP per worker—differs considerably between these countries. In 2015, it ranged between $34,920 (2011 PPP constant international dollars ($)) in Tunisia and $57,476 in Turkey (World Bank 2016).

In terms of demographic behaviour, these countries are expected to continue experiencing negative net migration with more people emigrating than immigrating (see UNPD 2016). Regarding fertility, the scenario assumes a continuation of past trends and that levels settle at the replacement level, but not before 2050. Regarding mortality, improvements in life expectancy are expected to be lower than those in the next, more favourable, scenario.

The *open economies scenario* assumes a future dynamic and globalized world economy. Production and trade take place without import and export barriers, while capital, labour, technology and innovation can move freely around the globe. In this scenario, the focus of government policies is to ensure smooth operation of production and trade systems, observing internationally agreed rules. The main objective is to create an ‘enabling’ environment for economic actors, contributing to the growth of total production and output per worker. Market forces, with limited government interference, mainly determine employment issues. The traditionally high share of public sector employment declines to levels comparable to the lowest levels currently observed in OECD countries (e.g. Boudarbat 2008; OECD 2014). Being aware that firms and the labour force operate in a competitive environment, governments take on an active role in supporting initiatives that contribute to the maintenance of existing economic competitive edges, exploration of new production niches and development of human resources. This becomes visible in high investments in development and modernization of education and vocational skill infrastructures; development of talent, skills and entrepreneurship; and by removal of social and economic barriers to the blossoming of talent. The society is characterized by a spirit of openness, which is reflected in the rapid adoption of new technologies, management and working styles in the population, among others.

During the period 2015–2020, relatively high emigration is observed among high-skilled and educated young adults for whom yet insufficient high-quality and adequately paid jobs are available. High emigration is also present among under- and unemployed persons who are attracted by prospects of higher income, better living conditions abroad and presence of family and relatives who migrated before and can be of assistance before, during and after migration (e.g. Boyd 1989; Castles et al. 2014; World Bank 2009).

During the period 2020–2025, Morocco, Tunisia, Algeria and Turkey are expected to start blossoming, leading to lower emigration and higher immigration, including return migration and immigration of children of former citizens who were born and raised in countries of destination of their parents (e.g. De Haas 2011). This trend continues after 2025 leading to historically high numbers of immigrants and return migrants and relatively low numbers emigrating. This scenario foresees increase of intercultural contact between these countries and EU countries, leading to change in gender relations and removal of sociocultural barriers to female labour force participation (e.g. World Bank 2011). Health services are expected to continue improving contributing to fertility decline as unmet need of family planning largely disappears. Fertility decline is also furthered because couples adopt smaller family size preferences. The latter is associated with higher decision-making power of women in fertility matters, higher female educational attainment and higher female labour force participation (e.g. Bloom et al. 2009). Fertility and mortality decline is more rapid than in both other scenarios.

The *closed economies scenario* is an anti-globalization scenario. The scenario describes a future resembling the state-society social contract model adopted by most governments in the Middle East and North Africa after independence (e.g. Boudarbat 2008; Schiffbauer et al. 2015). This scenario is also assumed to apply to Turkey although the country has a different social, economic and political history than the other three countries. The social contract development model attributes great importance to the role of the state in redistributing wealth, promotion of equity and provision of welfare and social services. The government has a final say in all issues related to employment, wage rates and regulation of economic markets. Citizens accept restrictions on political participation in exchange for economic security and provision of social services, protection and other benefits. Management of the national economy is mainly done by the state, with strong reliance on state planning. Governments perceive the nation as an organic unity requiring precise steering and monitoring to preserve unity and prevent social and political unrest.

Instead of economic collaboration with other countries, governments focus on protection of national markets from global competition and promotion of import-substitution industrialization. This inward orientation constrains the economy because it becomes difficult and costly to enhance worker productivity using new ‘foreign’ technologies and innovations. To increase production, firms have little choice other than hiring more local labour and extend and intensify the use of existing production equipment and technologies.

To cope with working-age population growth, governments permit institutions to expand staff, even if productivity is low, and they facilitate emigration (Ianchovichina and Lundstrom 2009; Schiffbauer et al. 2015). Up to the 1990s, a similar kind of development model was adopted in Maghreb countries, notably in Algeria. There, it led to considerable GDP growth rates, averaging 3.7% per year, contributing to decline of poverty, increase of health status, life expectancy, school enrolment and literacy. In later years, this development model proved to be too costly leading to lower GDP growth, stagnating output per worker and rising unemployment. Although economic stabilization and structural adjustment programmes of the International Monetary Fund (IMF) were adopted to revitalize the economy and restructure public sectors, implementation has been uneven, hesitant and incomplete (Schiffbauer et al. 2015).

This scenario therefore foresees that the social contract development model will prevail in the future. Although traditional destination countries increasingly restrict immigration, emigration is expected to increase to historically high numbers in these four countries because employment conditions do not improve and citizens find ways to realize their emigration plans using legal or illegal channels, including orientation on new destination countries. Immigration and return migration reduce to low numbers.

Low economic growth and rising public sector costs reduce the speed of fertility decline as governments spend less on maintenance and coverage of health and family planning services, leading couples to have more children than they actually want. Morbidity and mortality rates are also affected negatively so that life expectancy increases less rapidly than in the other scenarios.

### Operationalization

We used demographic and economic data compiled by international organizations. Demographic data were retrieved from databases and publications of the United Nations (UN) and World Health Organization. For instance, for the scenarios, we use base-year 2015 age-sex distributions of the UN Population Division (UNPD). These are derived from recent national census population counts and auxiliary demographic data sources (e.g. Demographic and Health Surveys) (UNPD 2016; WHO 2014). To the extent that data or estimates of temporary residents such as refugees and transit migrants are available, they have been included in UN mid-year 2015 population estimates, as such estimates are based on de facto and not on de jure residents. We realize that data on such kind of persons are known to be deficient so that they are most likely underrepresented in these population estimates. Economic data and indicators were derived from the World Bank (World Bank 2016). We formulated scenario assumptions of fertility, mortality and migration for the period 2015–2050 and also produce projection results for that period. As we choose for our economic scenarios a time horizon up to 2035, we only use the population scenario results up to 2035. Estimates of future size and age structure of the population, including working-age population estimates, were derived from demographic scenarios using the cohort component projection method (e.g. Preston et al. 2001). The quantitative assumptions of indicators of demographic and economic model variables, described below, are summarized in Table 1.

**Table 1:** Summary of scenario assumptions for indicators of model variables

Model variables and indicators		Business as usual (BAU)	Open economies	Closed economies
Population	Age distribution, by sex	UN 2015 base year	UN 2015 base year	UN 2015 base year
Migration	Net numbers of migrants, by sex	UN medium variant net migration numbers (negative for all four countries)	2015–2020 = 1.25 × average BAU level 2020–2025 = changes to average BAU level 2025–2030 = changes to zero by 2030 2030–2050 = changes to highest recorded net migration number, (+) sign is substituted for (−) if highest number is negative (−)	2015–2025 = average BAU level 2025–50 = changes to highest recorded *negative* net migration number
	Modes of change		Linear	Linear, constant
	Age pattern of migration, by sex	Model Western Standard UN (1992)^a^	Model Western Standard UN (1992)	Model Western Standard UN (1992)
Fertility	Total fertility rate (TFR)	TFR 2015 level changes to 2.1 by 2050	TFR 2015 level changes to 1.5 by 2050	TFR 2015 level changes to 2.4 by 2050
	Mode of TFR change	Linear	Linear	Linear
	Age pattern of fertility	UN medium variant	UN medium variant	UN medium variant
Mortality	Life expectancy at Birth e(0), by sex	0.1250 life expectancy years increase per calendar year	0.1875 life expectancy years increase per calendar year	0.0625 life expectancy years increase per calendar year
	Mode of e(0) change	Linear	Linear	Linear
	Age pattern of mortality, by sex	Constant WHO 2008 age pattern of mortality estimate by country	Constant WHO 2008 age pattern of mortality estimate by country	Constant WHO 2008 age pattern of mortality estimate by country
Economic production	GDP	2015–2035 = average annual GDP growth rate 2000–2015	2015–2035 = 1.25 times BAU scenario	2015–2035 = 0.5 times BAU scenario
Workers productivity	GDP/*W*	2015–2035 = average annual GDP/*W* growth rate 2000–2015	2015–2035 = 1.25 times BAU scenario Morocco (Morocco, Algeria), 1.5 times BAU scenario (Tunisia, Turkey)	2015–2035 = no increase, no decrease

^a^United Nations (UN) (1992), Preparing migration data for subnational population projections, pp. 41–44. New York. 1992

#### International migration

As explained before, Bruni and Venturini (1995) distinguish between migration pressure, measured as excess labour force or size of the non-employed population, and actual migration. The former is determined by macro-level economic factors while actual emigration and immigration is determined by micro-level factors where migration depends on a person’s propensity to migrate. Thus, it is possible that in a positive economic growth scenario migration pressure decreases because employment opportunities increase, while actual emigration also increases. The latter may occur if the propensity to emigrate rises. Therefore, migration assumptions for population scenarios up to 2050 do not conflict with inferences derived from results of those population scenarios regarding migration pressure (i.e. changes in the size of the non-employed population). Since immigration and, in particular, emigration statistics are often unreliable, we use migration assumptions expressed in net migration numbers. Baseline and historical net migration estimates were obtained from databases maintained by the UN (e.g. Preston et al. 2001). For the *business-as-usual scenario*, we adopted the United Nations medium variant net migration assumptions (UNPD 2016). The *open economies scenario* assumes higher net negative net migration up to 2020 than in the business-as-usual (BAU) scenario, as emigration is expected to increase in the take-off stage of development while immigration hovers around a constant low level. Between 2020 and 2025, growth of emigration is expected to level off and decrease when production and numbers of jobs start increasing. Return migration and immigration also increases, so that by 2025, negative net migration is at the BAU level, and by 2030, net migration has become zero. Between 2030 and 2050, net migration turns positive, and by 2050, it has reached a historically high (positive) level. The reason for this pattern of change is that, initially, during the take-off stage of economic growth, it takes time before sufficient numbers of quality and adequately paid jobs are generated which make many prospective migrants decide to stay and attract immigrants and return migrants. The *closed economies scenario* assumes that emigration increases while immigration and return migration become very low leading to an increase in negative net migration after 2025 to a historically high level of negative net migration number by 2050. In all scenarios, net migration numbers are redistributed to sex and age groups according to model age schedules of migration, while change over time is assumed to unfold in a linear way (Rogers and Castro 1981; UN 1992).

#### Fertility

The *business-as-usual* scenario assumes that average fertility will have settled at the replacement level by 2050 (i.e. total fertility rate (TFR) of 2.1 births). The *open economies* scenario assumes a somewhat larger decrease of fertility so that by 2050 the TFR will have declined to 1.5 births. The *closed economies* scenario assumes that average fertility will settle at an average of 2.4 births by 2050. All scenarios assume linear change in fertility levels while age patterns of fertility conform to those of the United Nations medium variant projections (UNPD 2016).

#### Mortality

The *open economies* scenario is the most favourable scenario in terms of health and mortality conditions. It assumes that the rate of increase in life expectancy in these countries is 75% of the average rate of increase—i.e. 2.5 life expectancy years per decade (Oeppen and Vaupel 2002)—observed in countries of the world where historical rates of increase have been highest. The *business-as-usual* and *closed economies* scenarios assume 50 and 25% of the Oeppen and Vaupel (2002) estimate, respectively. The World Health Organization (WHO) 2008 age pattern of mortality is assumed to apply in all scenarios (WHO 2014).

#### Gross domestic product

We assume that in the *business-as-usual* scenario the average annual GDP growth rate equals the average annual growth rate during the period 2000–2015. In all scenarios, we assume that the growth rates in the periods 2015–2025 and 2025–2035 are equal. For the *open economies* scenario, we assume that average GDP growth rates will be 1.25 times higher than those in the business-as-usual scenario in both time intervals, while the *closed economies* scenario assumes that GDP growth rates will only be half the rate assumed by the business-as-usual scenario.

#### Output per worker

The *open economies* scenario assumes that the growth of worker productivity will be higher than that in the *business-as-usual* scenario, while for the *closed economies* scenario it is assumed that up to 2035 worker productivity growth will be 0% per annum. The rationale for these assumptions is that the open economies scenario assumes that GDP growth is realized mainly by worker productivity growth and much less by growth of numbers of job vacancies. For the closed economies scenario, we assume the opposite. The case of Algeria is a special one as worker productivity growth has been at very low levels in the past, while, periodically, considerable GDP growth was realized. This was the result of labour market interventions of the government creating more jobs to reduce the exodus of male residents in search for a job abroad (Furceri 2012). Contrary to the situation in other countries, recent trends in GDP and worker productivity growth of Algeria, basis for the business-as-usual assumptions, closely resemble assumptions of the closed economies scenario. This is reflected in similar assumptions about future trends in these indicators.

## Results

### Working-age population prospects

Regarding the first research objective about deriving economic-demographic scenarios up to 2035 for our four study countries, Table 2 summarizes the main results of demographic projections for each scenario.

**Table 2:** Estimates of working-age-, total- and non-employed population, by country (×1,000)

		Scenarios						
		Base year	Business as usual		Open economies		Closed economies	
		2015	2025	2035	2025	2035	2025	2035
Morocco	Working-age population	22,899	24,644	26,969	24,156	26,087	24,185	25,942
	Total population	34,378	37,262	39,903	36,104	38,021	36,821	38,686
	Working-age population share	66.6%	66.1%	67.6%	66.9%	68.6%	65.7%	67.1%
	Non-employed population	11,373	11,388	11,443	11,340	11,574	10,004	8173
Algeria	Working-age population	25,991	29,231	32,369	29,107	32,049	28,403	30,699
	Total population	39,667	43,357	47,131	42,633	45,866	42,406	45,042
	Working-age population share	65.5%	67.4%	68.7%	68.3%	69.9%	67.0%	68.2%
	Non-employed population	14,221	12,322	8,077	16,206	17,909	14,296	13,790
Tunisia	Working-age population	7,771	8.098	8,591	8,066	8,461	7,778	7,947
	Total population	11,254	11,975	12,570	11,810	12,218	11,566	11,682
	Working-age population share	69.1%	67.6%	68.3%	68.3%	69.3%	67.3%	68.0%
	Non-employed population	4,359	4,209	4,159	4,233	4,155	3,776	3,252
Turkey	Working-age population	52,542	57,686	61,162	57,798	60,936	57,038	60,070
	Total population	78,666	84,563	90,469	83,892	88,871	84,088	89,557
	Working-age population share	66.8%	68.2%	67.6%	68.9%	68.6%	67.8%	67.1%
	Non-employed population	26,594	26,708	27,145	24,725	24,725	25,401	21,497

Results indicate that total and working-age populations are expected to continue growing between 2015 and 2035. In 2015, the Turkish population of 79 million was of similar size as the populations of the three Maghreb countries together (84 million), and their working-age populations were about 53 and 57 million, respectively. Working-age populations are expected to grow to figures close to 61 million in Turkey and to 65 million in the Maghreb countries. Algeria is the Maghreb country with the largest working-age population and Tunisia the one with the smallest.

According to expectation, differences between population scenario estimates are not great because differences in fertility assumptions only affect working-age population size in the long term, while effects of assumed changes in mortality and migration are relatively small, compared with the effect of cross-country differences in age structure in base year 2015.

Scenario estimates of the share of the working-age population in the total population of Morocco, Algeria and Turkey indicate that, depending on the scenario, shares may still increase slightly up to 2035. In the case of Tunisia, the working-age population share seems to have reached its maximum value. This is the result of Tunisia being an early adopter of small family size preferences in the past leading to reduced growth of the working-age population relative to the population as a whole (notably in comparison with growth of the elderly, i.e. age groups 65+).

The scenario results also show that the size of the population in the age range 15-64 without employment is expected to remain the same or slightly decrease in Morocco, increase or decrease in Algeria and Turkey (depending on the scenario) and decrease in Tunisia.

After around 2035—results not shown—all scenarios for all countries predict that working-age population shares will decline, with the steepest decline in Tunisia, while the share of the elderly in the population increases. For instance, by 2050, the working-age population share estimate for Tunisia will be in the range of 62 and 64%, depending on the scenario.

### Migration pressure prospects

Regarding the second research objective about exploring what the implications of the population scenarios are for employment-related migration pressure, Table 3 shows that in all countries migration pressure in terms of average annual growth of the non-employed population has been increasing (+) considerably during the baseline period 2000–2015. According to all scenarios, and for all periods, migration pressure is expected to decrease in Tunisia during the period 2015–2035. Regarding the other three countries, it depends on the scenario whether migration pressure is expected to increase (+) or decrease (−). In the case of the closed economies scenario, migration pressure in all countries is expected to decrease during the period 2025–2035. In the case of the business-as-usual scenario, both Algeria and Tunisia migration are expected to experience migration pressure decline during the period 2015–2035.

**Table 3:** Scenario estimates of annual change in migration pressure, by country

		Scenarios						
		Reference period	Business as usual		Open economies		Closed economies	
		2000–2015	2015–2025	2025–2035	2015–2025	2025–2035	2015–2025	2025–2035
Morocco	GDP	4.5%	4.5%	4.5%	5.6%	5.6%	2.3%	2.3%
	Output per worker	2.9%	2.9%	2.9%	3.6%	4.4%	0.0%	0.0%
	Working-age population	1.7%	0.8%	0.7%	0.6%	0.8%	0.6%	0.7%
	Employment ratio	−0.1%	0.8%	0.9%	1.4%	0.5%	1.6%	1.6%
	Non-employed population	180,621	1,528	5,492	-3,287	23,356	−136,840	−183,178
	Migration pressure	+	+	+	−	+	−	−
Algeria	GDP	3.6%	3.6%	3.6%	4.5%	4.5%	1.8%	1.8%
	Output per worker	−0.3%	0.0%	0.0%	3.6%	3.6%	0.0%	0.0%
	Working-age population	2.0%	1.2%	1.0%	1.1%	1.0%	0.9%	0.8%
	Employment ratio	1.9%	2.4%	2.6%	−0.2%	0.0%	0.9%	1.0%
	Non-employed population	105,304	−189,876	−424,462	198,533	170,245	7,490	−50,579
	Migration pressure	+	−	−	+	+	+	−
Tunisia	GDP	3.2%	3.2%	3.2%	4.0%	4.0%	1.6%	1.6%
	Output per worker	1.9%	1.9%	1.9%	2.8%	2.8%	0.0%	0.0%
	Working-age population	1.5%	0.4%	0.6%	0.4%	0.5%	0.0%	0.2%
	Employment ratio	−0.2%	0.9%	0.7%	0.8%	0.7%	1.6%	1.4%
	Non-employed population	65,423	−14,978	−5,012	−12,578	−7,770	−58,295	−52,416
	Migration pressure	+	−	−	−	−	−	−
Turkey	GDP	4.0%	4.0%	4.0%	5.0%	5.0%	2.0%	2.0%
	Output per worker	2.2%	2.2%	2.2%	3.3%	3.3%	0.0%	0.0%
	Working-age population	1.8%	0.9%	0.6%	1.0%	0.5%	0.8%	0.5%
	Employment ratio	0.0%	0.8%	1.2%	0.7%	1.1%	1.2%	1.5%
	Non-employed population	430,897	11,468	−252,805	55,109	−241,963	−119,245	−390,439
	Migration pressure	+	+	−	+	−	−	−

To explain Table 3, we discuss the figures pertaining to Algeria in case of the open economies scenario for the period 2015–2025. That scenario foresees that GDP growth during that period will mainly come from growth of worker productivity rather than from creation of more job opportunities and hiring of more workers. Annual GDP growth is assumed to be 4.5% and annual growth of output per worker is assumed to be 3.6% so that 80% of GDP growth is assumed to come from productivity growth.

Thus, only 4.5% − 3.6% = 0.9 percentage point of annual GDP growth is assumed to come from the growth of job opportunities and workers filling those vacancies. However, with 1.1%, the scenario-predicted annual growth rate of the working-age population is higher than 0.9% so that, in principle, only part of the working-age population growth can be accommodated in terms of employment. The implication is that the Algerian employment ratio will change by 0.9% − 1.1% = −0.2 percentage point annually. In a situation whereby the working-age population is growing, as in our study countries, the implication of a declining employment ratio is that the size of the non-employed population (also see Table 2)—our indicator of migration pressure—will increase. In the case of Algeria, the expected increase is 16,204 persons annually.

Table 3 also shows that in several other instances the scenarios predict that employment ratios are expected to increase while the size of the non-employed population is also expected to increase, such as in all scenarios for Turkey for the period 2015–2025.

This happens as long as the annual increase in the number of employed persons is smaller than the expected annual change^1^ of the size of the working-age population. The combination of increasing employment ratios and increasing size of the non-employed population and migration pressure is also predicted for Morocco by the business-as-usual scenario for both periods and by the open economies scenario for the period 2025–2035. This is also predicted by the closed economies scenario for Algeria for the period 2015–2025.

According to the business-as-usual scenario, our middle-of-the-road scenario, working-age population growth in Turkey and Morocco will still cause migration pressure to increase in the short-term (2015–2025) in these countries, while in Algeria and Tunisia a decline is foreseen. In the case of Turkey and Algeria, the most optimistic scenario in terms of creation of jobs—the closed economies scenario—an increase in the number of job opportunities and workers filling those positions is insufficient to accommodate working-age population growth. As discussed before, to what extent estimates of future change in numbers of non-employed will lead to change in actual migration depends on how propensities to migrate at the level of the individual may change in future (Bruni and Venturini 1995, pp. 380–381).

**Fig. 1:**
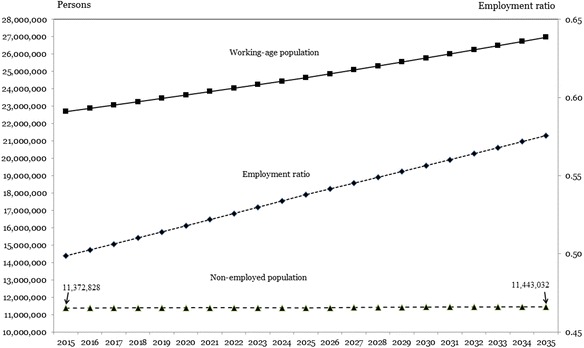
Migration pressure prospects for Morocco, business-as-usual scenario

Figure 1 illustrates how migration pressure in Morocco is expected to increase if the business-as-usual scenario would unfold. The figure shows how the working-age population grows at a rate of 0.8 and 0.7% annually during the 2015–2025 and 2025–2035 periods, respectively. Because of the assumed annual rates of increase of GDP and worker productivity, specified in Table 3, numbers of jobs are expected to increase each year.

The increase is such that the ratio of the number of workers to the total working-age population, i.e. the employment ratio, increases. However, the annual increase in jobs is smaller than the number of persons with which the working-age population grows each year. As a result, each year, a residual excess labour supply is added to the non-employed population (e.g. see Table 2) so that that the non-employed population is still growing each year, despite increase of the employment ratio. The conclusion therefore is that migration pressure for Morocco for the period 2015–2035 continues to increase.

**Fig. 2:**
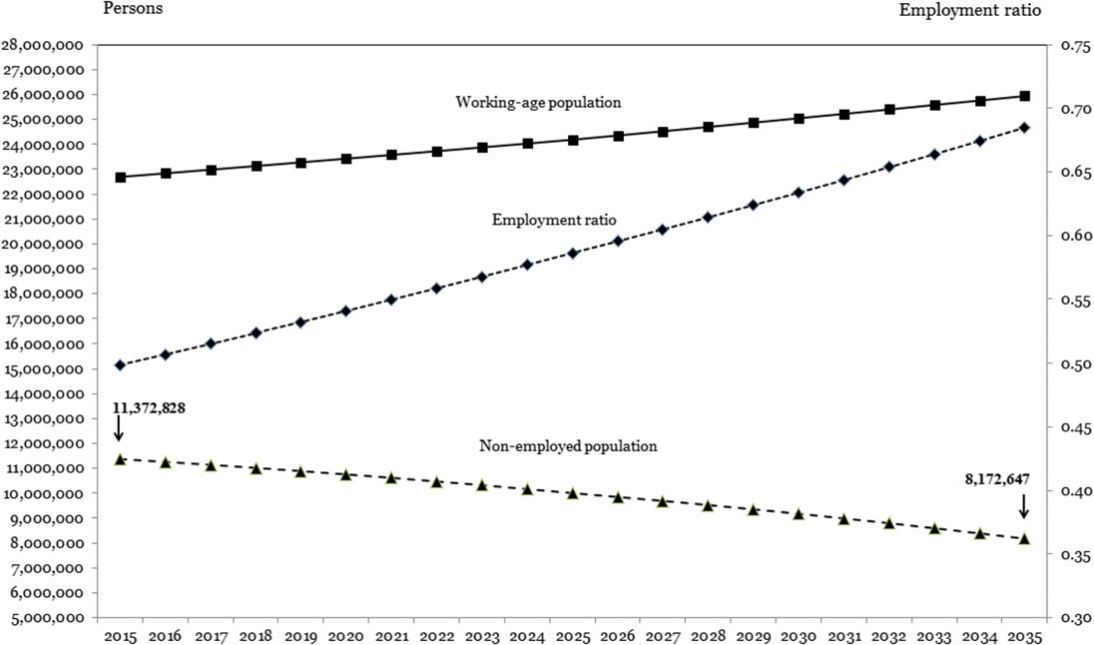
Moroccan migration pressure prospects, closed economies scenario

Figure 2 illustrates the migration pressure prospects for Morocco in the case of the closed economies scenario for the period 2025–2035. According to that scenario, in comparison with the business-as-usual scenario, a relatively larger part of the GDP results from increase of numbers of jobs rather than worker productivity. This can be verified from Table 3. The difference between the GDP growth rate and the worker productivity growth rate in the closed economies scenario (i.e. 2.3% − 0.0% = 2.3%) is larger than the difference between these two indicators in the business-as-usual scenario (4.5% − 2.9% = 1.6%). Furthermore, working-age population growth in the former scenario (e.g. 0.6%) is smaller than that in the latter scenario (e.g. 0.8%). The net effect is that the employment ratio is increasing to a much higher level by 2035 in the closed economies scenario than in the business-as-usual scenario. Annual numbers of jobs created exceed by far the annual number of persons with which the working-age population grows. Under the assumption that all job vacancies will always be filled, the annual shortage of number of workers, i.e. the positive balance of demand and supply of workers, will be recruited from the accumulated reserve of the non-employed population (also see Table 2). As a result, the size of the non-employed population—the excess labour in the economy—is shrinking rapidly each year, implying a rapid decrease of migration pressure.

If the past trend of the decline of population growth in these countries continues, with Tunisia as forerunner, it can be expected that, under conditions of the assumed (constant) economic growth indicators, more and more members of the working-age population will be absorbed by the economy. In the long term, this can be expected to lead to a decline of the size of the non-employed population and, thus, of migration pressure in these four countries.

## Discussion

In this article, we studied the migration pressure prospects for Algeria, Morocco, Tunisia and Turkey up to 2035. We explored prospects of economic and working-age population growth and implications for labour migration pressure. We defined labour migration pressure according to Bruni and Venturini (1995), which is excess domestic labour supply—measured as size of the non-employed working-age population—in the presence of negative per capita income differences with other countries. A parsimonious decomposition model was developed serving as analytical framework for the design and calculation of three economic and demographic scenarios. From the scenario results, estimates of future change in employment ratios and size of the non-employed population were derived for each country to explore the change in migration pressure during the period 2015–2035. The business-as-usual scenario describes a future that is a continuation of past trends in indicators of economic and demographic growth and of increasingly restrictive (EU) migration policies. The open economies scenario describes a future of an open and globalized world characterized by the free movement of persons, capital, innovation and technology and without migration restrictions. The closed economies scenario is a kind of anti-globalization scenario, including restrictive emigration and immigration policies.

Main findings are that, in spite of considerable differences in scenario assumptions, employment ratios are generally expected to increase so that relatively more persons of working-age will be employed in the future, a first sign of declining migration pressure in the future. However, the conclusion is also that the business-as-usual and open economies scenarios suggest that migration pressure in Turkey, and to a lesser extent in Morocco, is still expected to rise up to 2025 because the size of the non-employed population will still increase each year, though the speed of growth is decreasing. This counterintuitive situation occurs because the expected annual growth of numbers of jobs is outperformed by growth of the size of the working-age population. The fact that the direction of change in employment ratios and change in non-employed population size do not run parallel is important to policymakers because it may be tempting to conclude that policies resulting in lower unemployment rates (i.e. higher employment ratios) automatically lead to fewer people without employment. The numbers of non-employed may continue to rise at times when employment ratios rise. The expected higher employment-related migration pressure in most of these countries should be taken as an early warning for future higher and perhaps unwanted immigration into EU countries. To anticipate this, EU countries should, more than before, invest in reducing the migration pressure in the sending countries by assisting in tackling drivers of migration pressure, such as disparities with EU countries in terms of economic growth, employment, income, political stability and security (e.g. Borjas 1999).

We developed distinct population scenarios for our study countries while there are also readily available population scenarios for these countries. For instance, the IIASA 2010–2100 population scenarios by age, sex and educational attainment are available for 195 countries. They were derived within the context of five Shared Socioeconomic Pathways (SSP) scenarios of the Intergovernmental Panel on Climate Change (IPCC) (O’Neill et al. 2015; O’Neill et al. 2014; Samir and Lutz 2014; Samir et al. 2013). The five scenarios are storylines of different environmental and socioeconomic pathways to the future with distinct effects on emissions and climate change at the global level. Within the context of each SSP scenario, three different population scenarios are derived whereby assumptions about future levels of fertility, mortality and migration depend on whether a country already has low or high fertility rates or is a rich OECD country. In our opinion, the results of such population scenarios are not really suitable because they need to relate and to be fine-tuned to the particular research objective at hand. Ours is identifying and exploring different employment-related migration pressure trajectories, taking account of particular characteristics of the spatial context (i.e. the Mediterranean region and Europe). This is different from the IPCC SSP scenarios which explore different emission and climate change trajectories at the global level. Difference in research objectives and spatial context impinges on scenario-specific assumptions about drivers of demographic change (i.e. fertility, mortality and migration), and this may lead to different population scenario results.

Another issue that can be raised is whether we could have developed a more complex model which would better represent reality. Such a model could, if data permit, include labour force participation rates by gender and distinguish between full-, part-time- and underemployment, and by quality of employment, and by economic sector (Gutierrez et al. 2007). However, added model complexity would complicate interpretation of scenario results in light of employment-related migration pressure.

A few words need to be said about the plausibility of our scenarios.

Regarding the population scenarios, these cover years (2015–2020) when several countries in the Mediterranean region are going through a period of political and social transition of which the outcome is yet uncertain. We assumed that by 2025 this transition will have been completed resulting in a new status quo which does not lead to major shifts in demographic behaviour. However, is this reasonable to assume? What would happen in terms of demographic behaviour if Arab Spring-related protests would lead to the establishment of anti-western, conservative governments in these four countries? Can the presented population scenarios encompass working-age population growth trajectories emerging out of such kind of context? We think the answer is affirmative for the following reasons. The first and main reason is that the ‘population momentum’ embedded in age-sex pyramids in base year 2015 leaves little room for effects of changes in, say, fertility and mortality, due to a political reorientation between 2015 and 2035. For instance, regarding the impact on the working-age population, change of fertility rates during the period 2015–2025 will only become visible after 2035. The second reason is that effects of changes in fertility and mortality are attenuated because to some extent they cancel out such as in the case of higher fertility rates and lower life expectancies.

Regarding the economic scenarios, we assumed GDP growth rates in the range of 1.6 to 5.6%. Assuming a constant annual growth rate of 1.6% is indeed low growth as this figure would take an economy about 43 years to double its current GDP, while doubling time would be about 12 years if annual growth would be 5.6%. In light of the observed current low GDP levels of these countries, this seems as a plausible range for GDP scenario assumptions. Regarding worker productivity, we assume zero improvement or an improvement of max. 1.5 times of the observed worker productivity over a period of 10 years. Such kind of change has been observed in the past by these countries (ILO 2015).

We assume that migration pressure decreases if the non-employed population size decreases. In a few cases, we project a considerable increase of the employment ratio, but can employment ratios rise without constraints in these countries? For instance, in the case of Algeria, the business-as-usual scenario predicts that, by 2035, the employment ratio is expected to increase from about 45% of the working-age population in 2015 to 75% in 2035. Is such a rise realistic in a country where, in 2015, the male employment ratio is already at an intermediate to high level (75%) but where the female employment ratio is among the lowest in the world (14%) due to persistent and pervasive cultural barriers to work outside the home (Achoui 2006; ILO 2015; World Bank 2011)? The answer is affirmative if, during the period 2015–2035, cultural change permits higher female labour force participation. A rise of the general employment ratio to 75% by 2035 would imply the following. First, the male employment ratio would have to rise to even higher levels, say to 90%. Such a level seems feasible as it is observed in parts of Sub-Saharan Africa and Asia where the highest male employment ratios are found. Second, the employment ratio of Algerian women would then have to increase from 14% in 2015 to 60% by 2035 to realize a general employment ratio of 75% by 2035. Should existing barriers to female labour force participation prevail, the shortage of labour would then have to come from immigration, for instance, from Algerian labour migrants abroad. If this does not take place, a decline of GDP growth is implied.

To conclude, scenario results show that the tempo of migration pressure growth will level off because working-age population growth is becoming less strong. This supports claims of several qualitative and theoretical studies (e.g. De Haas 2011; OECD, 2009) which suggest that, in the long term, demographic growth will become less of a determining factor in the emigration of residents from these countries. However, up to 2035, the size of the non-employed population in Turkey and Morocco is expected to be large and even somewhat increasing. Proximity, presence of a large community of co-ethnics, political stability and safety and perceptions about better income and employment in EU countries may result in release of part of this migration pressure in the direction of Europe, through legal or illegal channels. If current political instability, sectarian violence, social unrest and insecurity in several countries in the South and East Mediterranean region continue to spill over to these countries, current refugee flows to the EU may not come to a halt and it will become increasingly difficult to distinguish asylum seekers from immigrants driven by economic motives. If so, acculturation of immigrants and social cohesion in EU countries and cities require even greater attention, anticipation, creativity and action of national policymakers and ethnic community leaders.

## Footnotes

1 Change in GDP, output per worker and working-age population is reported in terms of the average annual percentage growth in the 10-year time interval. Change in the employment ratio is reported as the average annual percentage-point change. Change in the non-employed population is reported as the average annual number of persons with which the non-employed population changes. The “+” or “−” signs pertaining to migration pressure conveys whether the non-employed population—our measure of migration pressure—is expected to increasing or decrease.
